# Genomic Characterization and Functional Description of *Beauveria bassiana* Isolates from Latin America

**DOI:** 10.3390/jof9070711

**Published:** 2023-06-29

**Authors:** Stefany Solano-González, Ruth Castro-Vásquez, Ramón Molina-Bravo

**Affiliations:** 1 Laboratorio de Bioinformática Aplicada (LABAP), Escuela de Ciencias Biológicas, Universidad Nacional, Heredia 40104, Costa Rica; 2 Centro de Investigación en Biología Celular y Molecular, Universidad de Costa Rica, San José 11501, Costa Rica; ruth.castrovasquez@ucr.ac.cr; 3 Biotecnología Vegetal y Recursos Genéticos para el Fitomejoramiento (BIOVERFI), Escuela de Ciencias Agrarias, Universidad Nacional, Heredia 40104, Costa Rica; ramon.molina.bravo@una.cr

**Keywords:** *Beauveria bassiana*, phytopathogen, fungal metabolism, CAZYmes, secondary metabolites, pathogenicity, functional genomics

## Abstract

*Beauveria bassiana* is an entomopathogenic fungus used in agriculture as a biological controller worldwide. Despite being a well-studied organism, there are no genomic studies of *B. bassiana* isolates from Central American and Caribbean countries. This work characterized the functional potential of eight Neotropical isolates and provided an overview of their genomic characteristics, targeting genes associated with pathogenicity, the production of secondary metabolites, and the identification of CAZYmes as tools for future biotechnological applications. In addition, a comparison between these isolates and reference genomes was performed. Differences were observed according to geographical location and the lineages of the *B. bassiana* complex to which each isolate belonged.

## 1. Introduction

Within *Beauveria*, about 25 species have been described, most of which originated in Asia—the species’ center of origin [1]. *Beauveria bassiana* (Bals.) Vuill (Ascomycota, Hypocreales) is a versatile microorganism capable of killing insects, surviving as a saprophyte in soil, and creating symbiotic associations with plants as an endophyte [2,3,4]. *Beauveria bassiana* sensu lato has a worldwide distribution and can infect around 700 insect species [5]. Since the discovery of its effect on silkworms [6], *Beauveria* has been used in the biological control of numerous agricultural pests [7,8].

According to phylogenetic studies based on the nuclear ribosomal internal transcribed spacer (ITS) and the elongation factor 1-alpha (TEF1-a) gene, *B. bassiana* has a monophyletic origin [9]. Within the species, many lineages have been identified and linked to a specific geographic distribution [10]. However, among strains of the same lineage, genetic variation is not directly associated with abiotic or biotic factors [10]. The same phenomenon has been reported for *Metarhizium anisopliae* (Metschn.) Sorokin [7].

Currently, there are 17 fully sequenced genomes of *B. bassiana* from Europe, Asia, Oceania (Australia), and North (USA) and South America (Colombia) available in the GenBank database of the National Center for Biotechnology Information (NCBI, https://www.ncbi.nlm.nih.gov/genome/browse/#!/eukaryotes/910/ (accessed on 16 May 2023)). More recently, eight additional genomes from Central America and the Caribbean, specifically Costa Rica, Honduras and Puerto Rico, have been added to the database [11], for a total of 25 sequenced genomes. Different levels of virulence and entomopathogenic responses have been linked to genetic variations, such as non-synonymous changes (NSCs) and copy number variations (CNVs) in important genes [12].

Costa Rica has implemented entomopathogenic fungi such as *B. bassiana* for the biological control of different insect pests [13] since the 1950s. National entities have made significant contributions regarding sampling, storing and characterizing fungal strains, such as The Tropical Agricultural Research and Higher Education Center (CATIE), the National University of Costa Rica (UNA), and the Cellular and Molecular Biology Research Center (CIBCM) at the University of Costa Rica (UCR) [11,14,15]. The work described herein analyzed eight of these isolates. One of these isolates (B01) has shown entomopathogenic activity against *Zabrotes subfasciatus* (Boh.) [16], while four other isolates (B13, B27, B43, B44) were extracted from infected *Galleria mellonella* (L.); all remaining isolates were extracted from soil in different agricultural systems [15]. However, this is the first study on the genomic characterization, comparison, and metabolic potential of *B. bassiana* fungal accessions from Central America (Costa Rica, Honduras) and the Caribbean (Puerto Rico). Our results provide new insight for the understanding of the pathogenic capacity of the species and the creation of a possible global metagenome. We have identified interesting genetic elements that potentially contribute to the pathogenicity as well as other interesting enzymes that add value to the understanding of the entomopathogenic activity of *B. bassiana*. Here, we report fungal isolates from the neotropics and provide an overview of the genomic features targeting pathogenic genes and their secondary metabolism potential. We also provide a comparison between these isolates and the previously characterized reference genomes.

## 2. Materials and Methods

### 2.1. Fungal Isolates and Genomic Material

The fungal isolation, DNA extraction, library prep and sequencing strategy was previously reported for eight *B. bassiana* isolates [11]. A simplified nomenclature was used herein, whereby BV-ECA# was replaced with B# (B0, B1, B13, etc.).

### 2.2. De Novo Genome Assembly Process

Prior to the de novo genome assembly, raw Illumina reads were quality-checked using FastQC [17] and processed using Trimmomatic v.0.38 [18] to remove the sequencing adapters and quality reads below a cutoff of 30. Quality passed reads were used for the de novo assembly using SPADES v.3.13.1 [19,20] at the default settings, except for the kmer size values, which were set as 89, 95, 97, 101, 107, 117 and 127.

Genome assemblies were compared and evaluated using Quast v.5.0.2 [21] against the *Beauveria bassiana* ARSEF 2860 (gb|ADAH00000000.1) reference. To identify the kmer’s distribution through the genomes and to plot its distribution, Jellyfish v.2.2.10 [22] and GenomeScope [23] were used, respectively. This allowed us to rapidly determine the overall characteristics of the genomes, regarding genome size, heterozygosity rate and repeat content.

Due to the short-length nature of Illumina reads and the intrinsic complexity of producing high quality eukaryotic genome assemblies in one go, we implemented the Multi-Draft based Scaffolder (MeDuSa) to optimize the draft assembled genomes scaffolder Medusa v.1.6 [24] against a set of assembled *B. bassiana* genomes: *B. bassiana* strains ARSEF 2860 (gb|ADAH00000000.1); Bv062 (gb|GCA_003337105.1); *B. bassiana* JEF-007 (gb|GCA_002871155.1); and *B. bassiana* HN6 (gb|GCA_014607475.1). To assess the quality and completeness of the assemblies, Quast v.5.0.2 [16] and BUSCO v. 3.0.1 [25] were used with both the eukaryotic (eukaryota_odb9) and fungal (fungi_odb9) datasets.

### 2.3. Gene-Calling and Identification of Putative Secreted Proteins

As a first proteome draft, proteins were predicted by implementing Augustus v2.6.1 [26] trained on *Aspergillus oryzae*, due to its well-documented necrotrophic lifestyle. In order to identify putative secreted proteins within each fungal isolate, SignalP 4.0 [27] was run locally; predicted proteins were considered to be secreted if SignalP identified a secretion signal peptide and no transmembrane domains. 

### 2.4. Fungal Phylogeny and Comparative Genomics

A phylogeny tree was constructed using single-copy BUSCO genes [25] for all *B. bassiana* isolates [11] and reference *B. bassiana* strains ARSEF 2860 (GenBank accession no. ADAH00000000.1); *B. bassiana* HN6 (GenBank accession no. GCA_014607475.1); and *C. militaris* CM01 (GenBank ID no. GCA_000225605.1). Single-copy BUSCO genes were identified within each organism and their corresponding proteins were aligned with Multiple Alignment using Fast Fourier Transform (MAFFT) v7.397 [28] with the options “--maxiterate 1000–auto”, and each alignment was trimmed using TrimAl v1.4.rev22 [29] with the option “--gappyout”. For each of the 293 single-copy BUSCO genes from each species, we implemented IQTree v.2.2.0-beta software [30]. An automatic detection to identify the best-fitting model with the option “-m MFP” was used, which led to the best JTT + F + R model. To obtain the best-scoring ML tree, five independent tree searches were conducted with the option “--runs 5”; the topological robustness of each gene tree was evaluated using the option “-bb 10,000” with 10,000 ultrafast bootstrap replicates. *C. militaris* CM01 was used [31] as the outgroup of the tree. Orthofinder version 2.5.2 [32] aided the identification of unique genes and orthologous relationships between the proteomes from fungal isolates and the reference *B. bassiana* ARSEF 2860 through standard mode parameters.

### 2.5. Metabolic Pathways Description and Fungal Functionality

For the discovery of the potential biological meaning of the fungal isolates, the BlastKOALA tool was implemented as an automatic annotation server for the genome and metagenome sequences [33]. KEGG orthology assignments were performed with BlastKOALA to characterize individual gene functions and for the reconstruction of the KEGG pathways and modules in the fungal isolates. 

*B. bassiana* genome annotation for Carbohydrate-Active Enzymes (CAZymes) was performed automatically using the Augustus called-proteins through the dbCAN2 meta server [34] with the databases dbCAN, HMMM and CAZy for pattern recognition. Hits were considered when an annotation was present in all three databases. Additionally, antiSMASH [35] software version 6.1.1 was used locally to identify important gene clusters associated with secondary metabolites, such as NRPS, TPKS and Terpenes.

To identify the presence of potential virulence-associated genes, blast analysis was conducted against the host–pathogen interactions database (HPIDB 3.0) [36]. The Blosum 62 matrix was implemented and only genes that showed a percentage of identity greater than 70% and a coverage greater than 50% were selected. We searched for PHI genes involved in virulence, genes responsible for degrading insect cuticles, mating-type genes, and core genes involved in the biosynthesis of secondary metabolites. The pathogenicity or virulence reported for a particular fungus was presumed to be similar or identical in *B. bassiana*, as there are no entries for entomopathogenic fungi in the PHI-base [37]. Mating-type loci identification in *B. bassiana* isolates and fungal references were identified using homology to characterized MAT genes specific to *Clavicipitaceae* and *Cordyceps* sp. using BLASTp. Selected hits fulfill both an e-value of 0.05 or lower and a query coverage filter of at least 55 (blast command line options-qcov_hsp_perc 55-evalue 1 × 10^−5^).

## 3. Results

### 3.1. Genome Assembly and Completeness Statistics

Unprocessed short raw reads were analyzed using Jellyfish and GenomeScope to plot the kmer distribution to check for genome size, heterozygosity rate and repeat content. Globally we observed an optimal genome size, haploidy and low repeat content for all isolates (Supplementary Figure S1). 

The predicted proteome for each isolate, processed through Augustus v2.6.1 trained on the *Aspergillus oryzae* dataset, called out 9465 to 10,453 putative proteins. From these, isolates B0, B13, B26 and B27 presented similar counts for potential secreted proteins (1007, 1013, 1003 and 1012, respectively), strains B1 and B31 had 1028 and 1039, respectively. Similarly, strains B43 and B44 reported 1058 and 1061 (Table S1). 

### 3.2. Fungal Phylogeny and Comparative Genomics

**Figure 1:**
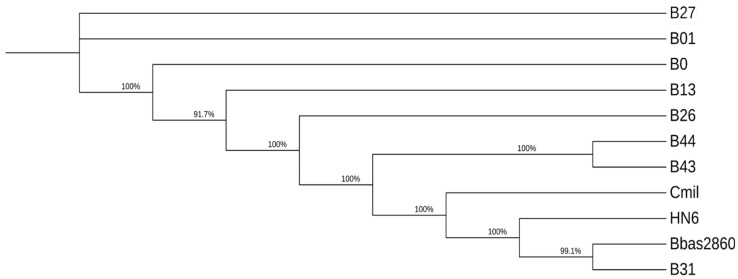
Maximum likelihood tree constructed from single-copy BUSCO genes for *B. bassiana* assemblies, with Bbas ARSEF 2860, BbasHN6, and *C. militaris* CM01 strains as references. This tree was constructed using MAFFT alignments of 2930 full single-copy protein coding sequences through IQ-Tree, implementing the JTT + F + R5 model and 10,000 replicates for ultrafast bootstraps support, depicted in percentages. The tree depicts the Costa Rican isolates (B0, B01, B26 B27 and B31), Honduran isolate (B13), and the Puerto Rican isolates (B43 and B44). Our results show high values for bootstrap and, interestingly, *C. militaris CM01*, considered as an outgroup clustered to *B. bassiana* references (HN6 and Bbas2860) and isolate B31.

To construct the phylogenomic analysis, as inputs, we used single-copy full-length BUSCO genes from all the *B. bassiana* sequenced isolates from this study; reference strains Bbas ARSEF 2860 (gb|GCA_000280675.1); BbasHN6 (gb|GCA_014607475.1); and *C. militaris* CM01 (gb|GCA_000225605.1) were included as outputs. This analysis produced a total of 293 core-genes. For each gene, its corresponding BUSCO protein was selected, concatenated and aligned using MAFFT, and an ML tree was inferred using IQ-Tree (Figure 1).

**Figure 2:**
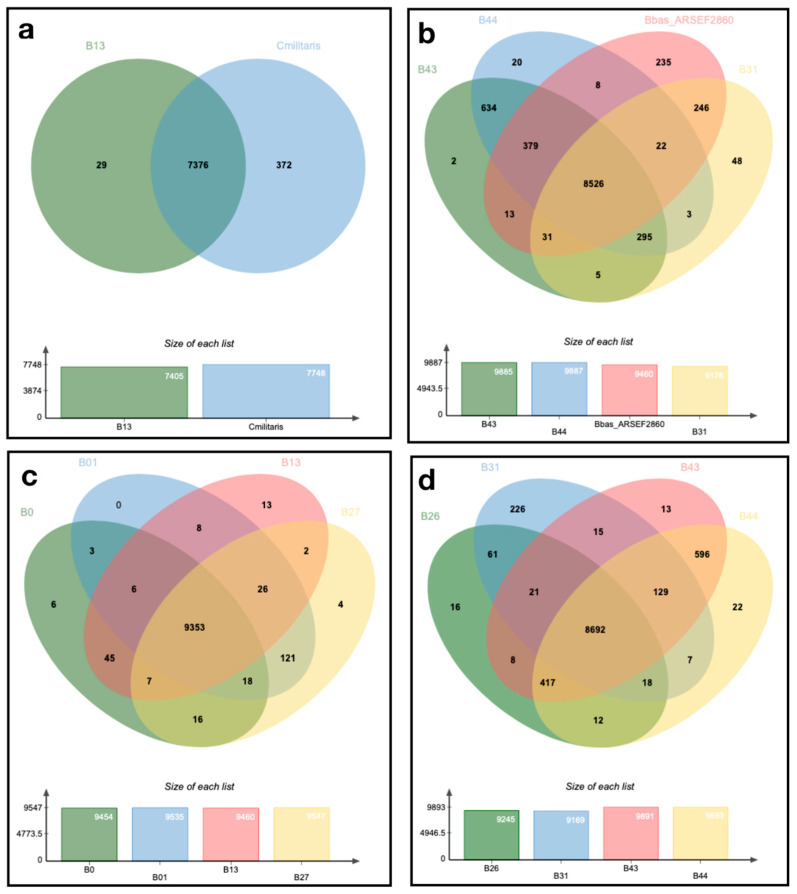
Venn diagram of unique and common orthologous gene clusters from *Beauveria bassiana* isolates, and *B. bassiana* ARSEF2860 and *Cordyceps militaris* CM01 references. Analysis based on Orthofinder outputs. (**a**) Genomic comparison for isolate B13 and C. militaris reference. (**b**) Genomic comparison for isolates B43, B44, B31 and B.bas ARSEF 2860 reference, (**c**) Genomic comparison for isolates B0, B01, B13 and B27, (**d**) Genomic comparison for isolates B26, B31, B43 and B44. All comparisons include graph bars illustrating overall genes counts for each organism.

The Orthofinder ortholog comparison between the fungal isolates revealed that 96,233 genes (98.1% of the total) were assigned into 10,356 orthogroups. Fifty percent of all genes were assigned in orthogroups with 10 or more genes (G50 was 10) and were contained in the largest 4546 orthogroups (O50 was 4546). There were 7047 orthogroups within all species and 6284 of these consisted entirely of single-copy genes. In addition, there were 1899 unassigned genes accounting for 1.9% of the total number of genes (Figure S1). All isolates shared a large number of orthologous genes, between 8526 and 9353, including the reference genome (ARSEF2860; Figure 2). Interestingly, isolate B31 shared the highest number of proteins with *C. militaris* CM01. Additionally, when in combination with the shared proteins among the genomes, B26 shared the lowest number of orthologous genes with other isolates (Figure 2).

### 3.3. Metabolic Pathways Description and Fungal Functionality

To introduce biological information using KEGG categories and the number of gene counts, we focused on specific functional categories (Table 1). B43 and B44 showed the closest amount of protein counts compared with the reference genome but B44 had a greater protein count than ARSEF 2860. On the other hand, B26 showed the lowest protein count with respect to the reference and the other isolates. All the KEGG terms, in general, were uniformly distributed among the isolates.

From our analysis, a total of 2349 protein models were assigned to CAZymes distributed into the Auxiliary Activities (AA), Carbohydrate Binding Modules (CBM), Carbohydrate Esterase (CE), Glycoside Hydrolase (GH), Glycosyl Transferase (GT), and Polysaccharide Lyases (PL) families. Most proteins (54%) were associated with the GH family, followed by the GT family (30%) and various other gene families (Table 2).

We focused on identifying key members from the GH family, which are involved in the enzymatic deconstruction of chitin [38] and harbor chitinases that are targets for the biological control and biotech industry. For each assembled and annotated *B. bassiana* isolate, we identified 18 putative glycoside hydrolase family 18 (GH18) CAZymes; whereas for the *C. militaris* CM01 and *B. bassiana* ARSEF2860 references, we identified 21 genes for each reference. Other important chitinases in the families GH3 and GH16 were absent in our isolates, while for the families GH75 (Chitosan) and GH76, two and nine enzymes were identified, respectively. Furthermore, family GH10 enzymes, present in our isolates, have only been reported in *Beauveria bassiana* [39] and are absent in other entomopathogenic fungi (*Cordyceps militaris* (L.), *Metarhizium anisopliae* and *Metarhizium acridum* (Driver & Milner) J.F. Bisch.), while the GH84 and GH95 families, which were reported previously as exclusive for entomopathogenic fungi [39], were observed in all the isolates included in this study. Families GH5_5 and GH45, which target cellulose, were also present in our data as in previous studies [39].

On the other hand, the CBM66 member from the Carbohydrate-Binding Module family is reported to be involved in binding fructans [40]. Interestingly, only *C. militaris* reported one combination of GH18 + CBM66 (Table S2). From the annotated GH18 subfamily, according to the CAZyme database, some enzymes report a characterized deconstruction of chitin. From these, the chitinase enzyme (EC. 3.2.1.14) represented 60% (112) of the annotated GH18 for all isolates, the unspecified glycosidase EC. 3.2.1.- represented 19.4% (33), while the enzyme mannosyl-glycoprotein endo-beta-N-acetylglucosaminidase (EC 3.2.1.96) represented 13% (23) (Table S3). From the same substrate-specific characterized enzymes, the chitinases EC. 3.2.1.96 and EC 3.2.1.17 were not identified.

**Figure 3:**
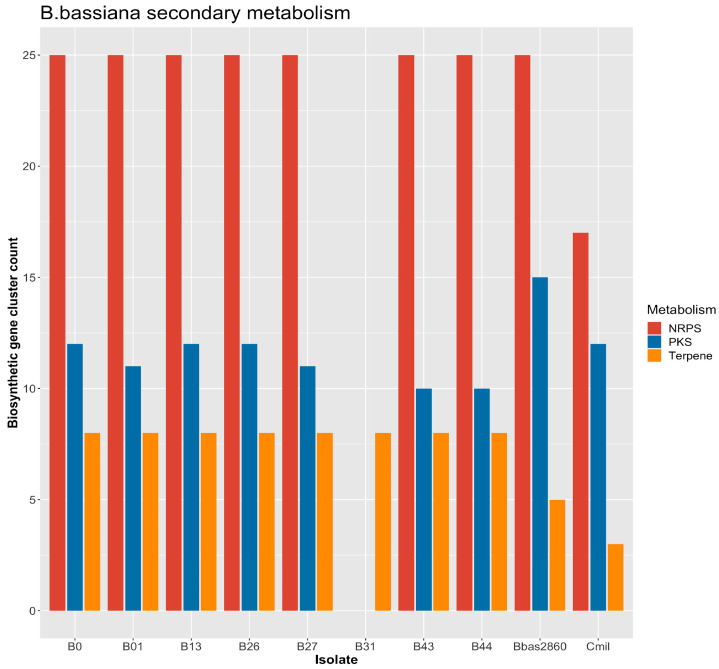
Nonribosomal peptides (NRPS), polyketide synthases (PKS), and terpene gene cluster counts for eight neotropical *Beauveria bassiana* genomes (B0, B01, B13, B26, B27, B31, B43, and B44) and two references: *B. bassiana* ARSEF8026 (Bbas8026) and *C. militaris* CM01 (Cmil).

In terms of secondary metabolite potential, the majority of the biosynthetic gene clusters were associated with Non-ribosomal Peptide Synthetases (NRPS), whereas those for Polyketide synthases (PKS) and Terpenes were comparable (Figure 3). All genomes had the same number of terpenes compared with ARSEF 2860 and *C. militaris*. However, in the B31 isolate, no NRPS and PKS were observed. The Puerto Rican isolates B43 and B44 consistently showed similarity throughout the analysis.

### 3.4. Identification of Functional Pathogenic and Important Virulent Elements and Mating-Type Genes

To identify the presence of potential virulence-associated genes, a blast analysis was conducted against the Pathogen–Host Interaction database v4.5 (PHIbase). Hits above 70% were the most informative and reliable. We identified PHI genes for important gene families, such as virulence-associated genes, degrading insect cuticles genes, mating-type genes, and core genes for the biosynthesis of secondary metabolites (Table 3). From this, we identified orthologues of the most important pathogenic genes [41]. Each gene was grouped into a single-copy gene orthogroup, except for the CYP52X1 and HSP90, as there are two copies for B26 and the Bbas reference, respectively. MrpacC is a transporter involved in ion transport and/or toxin secretion and was only present in the B0 and B1 isolates. Similarly, a hydrophobin (Hyd2), a protein involved in hydrophobic interactions on the insect cuticle, was identified in B0, B1 and B31 (Table 3).

Concerning mating-type genes, we compared the proteome for each *B. bassiana* isolate from this study against a database downloaded from the NCBI database specific to the Clavicipitaceae and *Cordyceps* species. This protein database contained 372 unique entries. We identified seven potential orthologues for each genome respective to the database. Interestingly, each gene was in a single-copy gene orthogroup, except for one orthogroup that lacked a protein for B31, and the genome references *B. bassiana* ARSEF 2860 and *C. militaris* CM01; this corresponded to a mating-type protein a-1 (orthogroup OG0009027). On the other hand, the six mating-type candidates had a corresponding single-copy protein in *C. militaris,* and all homologues showed a high level of conservation (Figure S3). 

Two orthologues coded for an HMG-box protein; members of this family include the fungal mating-type gene products MC, MATA1 and Ste11 [42]. The other orthologues coded for a DNA repair protein, rad10, DNA lyase, timeless protein and the fungal pheromone mating factor STE2 GPCR. This orthologue was missing in B31, and both references coded for the mating-type protein a-1. All the isolates showed similarity for the mating-type protein MAT1-2-1, except B31, which showed a MAT1-1-1 gene as the *C. militaris* CM01 and *B. bassiana* ARSEF 2860 reference genomes.

## 4. Discussion

*B. bassiana* isolates from different locations in the neotropics were previously sequenced and assembled [11]. For each isolate, Jellyfish and GenomeScope were implemented to plot the kmer distribution and to check for the genome size, heterozygosity rate and repeat content from unprocessed short reads. Although there are no *B. bassiana* reference genomes at BUSCO, Castro-Vásquez et al. [11] reported values ranging from 96% to 97% using the BUSCO eukaryota_odb9 database fixed for *Fusarium graminearum*. In the study herein, we additionally used the BUSCO fungi_odb9 fixed set for *Aspergillus oryzae*, which gave a variation percentage ranging from 88% to 98%, indicating lower universal single-copy orthologous genes found in the assembled genomes with the chosen training data set. According to Waterhouse et al. [43], BUSCO genes have been widely used as a measure of genome completeness, and additionally as markers for fungal phylogenomic relationship inferences [44,45]. Both datasets have different gene categories that made this analysis more robust due to the lack of specific *B. bassiana* databases.

Phylogenetic analyses performed in previous studies determined that all the isolates (B0, B1, B13, B26, B43, and B44), except for B27 and B31, belong to an African-Neotropical lineage of *B. bassiana* [15] defined by Rehner et al. [10]. In the analysis herein, of 293 core-genes, almost each isolate represented a particular cluster (B0, B1, B13, B26, and B27). Only the Puerto Rican isolates (B43 and B44) were together in the same cluster. On the other hand, B31 grouped with the reference *B. bassiana* and *C. militaris* genomes, corroborating that this isolate is genetically distinct from all the other isolates analyzed. Throughout several analyses, B31 had particular similarities with *C. militaris* (number of proteins, lack of the orthogroup OG0009027, same mating-type gene) and, at the same time, showed a complete absence of NRPS and PKS compared with the other isolates and reference genomes. In previous analyses, B31 also showed unique alleles in several SSR markers and its lineage could not be determined, while the rest of the isolates were highly diverse but, hence, distinct from *Cordyceps* [15].

The Orthofinder ortholog comparison between the fungal isolates and *B. bassiana* (ARSEF 2860, HN6) and *C. militaris* (CM01) reference genomes confirmed the singularity of the B31 isolate, whose proteome had a greater similarity to *C. militaris* than the *B. bassiana* references and isolates. The direct ancestors of *B. bassiana* were Asian *Cordyceps* species, according to Xiao et al. [1]. Additional evidence has also confirmed the link between *Beauveria* anamorphs and *Cordyceps* teleomorphs [46,47,48]. Among the principal differences between *B. bassiana* and the *Cordyceps* species is the capability of the former to infect a wide host range, while *Cordyceps* species usually manifest host specificity [1]. *Metarhizium* species have been shown to evolve from specific-host species to generalist-host species [49]. This raises an interesting question as to whether B31 represents an intermediate species between *Beauveria* and *Cordyceps* or a new lineage of the *B. bassiana* sensu lato complex. New species of *Beauveria* have been reported in South America [50,51,52] and more could still be found in the rest of the continent. Similar phenomena have been observed in Asia, where a notable abundance of newly identified species demonstrates high biodiversity. However, among these species, the prevalence of one or two *Beauveria* species, typically *B. bassiana*, tends to overshadow the presence of the newly determined species [53,54].

KEEG analysis of important metabolic pathways, such as the environmental information processing, biosynthesis of secondary metabolites, and lipid and amino acid metabolism, were completed in our genomes. Those pathways are related to the capability of the isolates to develop a pathogenic response and toxin production during the infection process [1]. Previous reports have established that the expression of those genes can be strain differentiated, and as a consequence, can have different environmental nutrition acquisition capabilities [55]. We also analyzed CAZyme genes due to their capabilities in degrading lignocellulose development and the stress response [39]. CAZymes have been reported for *B. bassiana* genomes and other entomopathogenic fungi [39], and herein, we provide further support for those findings as well as a list of potential genes that emphasize carbohydrate-active enzymes in tropical isolates of *B. bassiana***.** In the case of secondary metabolite precursors, the *B. bassiana* genome contains 45 SM core genes, including 13 NRPS, 12 PKS, 7 NRPS-like, 1 PKS-like, 3 hybrid NRPS-PKS, and 12 genes related to FAS/terpene/steroid biosynthesis [1]. A reduction in the NRPS and PKS genes in entomopathogenic species that cause systemic infection of host tissues, such as *Metarhizium acridum*, has been reported by Gao et al. [56]. The reason for the total lack of NRPS and PKS genes in the B31 isolate is unknown, but the differences between this isolate and the other isolates is consistent throughout several of our analyses. *C. militaris* contains enough genes for the infection of specific hosts, while *B. bassiana* and *M. anisopliae* are mostly generalist species and have the same NRPSs as the ARSEF2860 reference and fewer PKS components.

There are no entries for entomopathogenic fungi in the PHI-base [37], therefore we assumed that the proof of pathogenicity/virulence reported in one fungus would also suggest a pathogenicity/virulence function in fungi within our study. Some of the gene families and orthogroups identified showed differences. Hyd1 was present in all isolates, but Hyd2 was absent in five (B13, B26, B27, B43, and B44) out of the eight isolates. Previous studies have shown that Hyd1 has a greater role on virulence than Hyd2; the main phenotype effect of Hyd2, when absent, is reduced surface adhesion [57]. On the other hand, five ABC transporters have been examined in *B. bassiana* including one B-type, one C-type and three G-type, and only the C- and G-type showed decreased virulence in topical bioassays [58] (Liu et al., 2011). The MrpacC transporter was absent in most of the isolates except for B0 and B1, and this must have been in consideration during the selection of these isolates as potential biocontrollers.

MAT-type genes identified in our isolates indicated that all possess MAT1-2-1 genes except for B31, which contained MAT1-1-1 genes. In both cases, these are the genes present in *B. bassiana* genomes [1] and a third kind of gene has been reported in *B. bassiana* isolates, MAT1-2-8 [42]. Regardless of these mating-type genes present in *Beauveria* genomes, sexual reproduction is infrequent in nature, according to Xiao et al. [1]. The lack of important genes, such as Spo11, which are crucial for meiotic recombination during sexual reproduction, was reported by Xiao et al. [1] as a possible reason as to why asexual reproduction is common in natural populations of *B. bassiana*. However, Valero-Jiménez et al. [42] described the presence of this gene in five isolates of *B. bassiana* and all of the genomes described herein also have this gene. Can the preference for asexual reproduction in *B. bassiana* be an adaptation for prolific geographical expansion, or is it used to enable the infection of a wide range of hosts? Or, as reported by Xiao et al. [1], are transposable elements the main force introducing genetic variation and genome evolution in *Beauveria*? Furthermore, is this mechanism effective enough to avoid sexual reproduction? These are questions that need to be answered in future studies.

Since allopatric speciation was previously described by Rehner et al. [10] as an important force to introduce genetic differentiation in *B. bassiana* complex lineages, we analyzed our isolates’ unique genes, and found differences between the Puerto Rican isolates (B43 and B44) and other isolates, indicating a possible geographic differentiation. Similarly, continental isolates belonging to different lineages defined by Rehner et al. [10], such as B27 (North American lineage), showed 116 unique genes compared with B0 and B13 of the African and Neotropical lineages. We also observed a major differentiation in B31, whose lineage could not be determined; compared with the other isolates, a total of 533 unique genes were found in B31. High strain diversity has been reported in populations of entomopathogenic fungi around the globe, which indicates that complex interactions between abiotic and biotic factors are also important forces for gaining genetic variability at a local level [7]. According to Valero-Jiménez et al. [42], the Bb8028 isolate has 163 exclusive genes compared with four other isolates used for the control of malaria mosquitoes. Valero-Jiménez et al. attributed this genetic differentiation to the isolate’s association with host adaptation due to its high virulence effect in *Anopheles* vectors. Perhaps, due to B31’s association with Costa Rican sugarcane fields, a similar effect is occurring; however, further analysis is needed to investigate this pronounced genetic differentiation.

There are still countries, such as Belize, Guatemala, El Salvador, Honduras, Nicaragua, Panama, Dominican Republic, and Haiti, where there are no genomic studies or the molecular characterization of *Beauveria* or other entomopathogenic fungi. Studies from Mexico [59,60], Brazil [61] and Costa Rica [14,15,62], have shown high genetic diversity in *Beauveria* isolates. Untapped *Beauveria* diversity in the neotropics through genomics is crucial to develop a comprehensive international landscape of the *B. bassiana* complex and the different species of the genus. The above, including the finding of *Cordyceps* ancestor species, is needed to map their evolutionary relationships, host, niche and environmental adaptations.

## Figures and Tables

**Table 1:** Summary of the biological systems in eight *B. bassiana* neotropical isolates and the *B. bassiana* ARSEF 2860 reference. A distribution of the KEGG systems is displayed as a list. The number of gene counts is shown for each category. A total of nine KEGG terms are displayed.

	Fungal Isolate								
Feature	ARSEF 2860	B0	B01	B13	B26	B27	B31	B43	B44
Protein count	10,364	9593	9636	9566	9465	9648	9546	10,210	10,453
% annotated	38.1	39.3	39.2	39.4	39.8	39.1	39.7	37.5	37.5
Functional Category									
Genetic information processing	1623	1549	1556	1552	1552	1557	1553	1572	1588
Carbohydrate metabolism	309	297	298	297	298	299	299	301	313
Cellular and signaling processes	467	456	451	453	458	452	452	458	484
Environmental information processing	191	184	185	183	186	185	190	184	188
Amino acid metabolism	199	179	179	182	177	179	181	186	193
Protein families related to metabolism	167	160	162	161	159	162	167	171	170
Unclassified metabolism	166	151	152	150	150	153	154	155	158
Lipid metabolism	158	147	148	150	147	148	151	150	153
Energy metabolism	122	118	119	119	117	118	117	117	123
KEGG Modules	85	80	78	79	78	78	81	80	81
KEGG Pathways	405	402	402	402	399	492	400	401	406

**Table 2:** Putative CAZymes’ family domains distribution for *B. bassiana* isolates, and references *B. bassiana ARSEF* 8028 and *C. militaris*, corresponding to Auxiliary Activities (AA), Carbohydrate Binding Modules (CBM), Carbohydrate Esterase (CE), Glycoside Hydrolase (GH), Glycosyl Transferase (GT), and Polysaccharide Lyases (PL).

Individual Count	Fungal Isolate	AA	CBM	CE	GH	GT	PL
300	ARSEF 2860	41	2	5	164	87	1
293	B0	38	2	7	158	87	1
294	B1	38	2	7	158	88	1
294	B13	38	2	7	158	88	1
290	B26	38	2	5	158	86	1
293	B27	38	2	7	158	87	1
292	B31	38	2	6	161	84	1
291	B43	39	1	6	158	86	1
292	B44	39	1	6	159	96	1
290	*C. militaris*	36	2	9	158	81	4
	Total count *	306	14	51	1268	702	8
	%	13%	0.50%	2.17%	54%	30%	0.30%

* Includes only isolates from B0 to B44.

**Table 3:** Gene copies associated with pathogenicity functions in assembled and annotated *B. bassiana* genomes identified through PHI database analysis.

Pathogenic Gene	B0	B01	B13	B26	B27	B31	B43	B44	Bbas_ARSEF2860
BbCHIT1	1	1	1	1	1	1	1	1	1
BbMBF1	1	1	1	1	1	1	1	1	1
CYP52X1	1	1	1	2	1	1	1	1	1
Chi2	1	1	1	1	1	1	1	1	1
HOG1	1	1	1	1	1	1	1	1	1
HSP90	1	1	1	1	1	1	1	1	2
MrpacC	1	1	0	0	0	0	0	0	1
Hyd1	1	1	1	1	1	1	1	1	1
Hyd2	1	1	0	0	0	1	0	0	1
MrGAT	1	1	1	1	1	1	1	1	1
Pmr1	1	1	1	1	1	1	1	1	1

## Data Availability

All data analyzed in this study are cited and summarized in the manuscript.

## Supplementary Materials

The following supporting information can be downloaded at: https://www.mdpi.com/article/10.3390/jof9070711/s1, Table S1: SignalP gene IDs for each genome, individual gene numbers (per column). Table S2: Counts for putative CAZymes for *B. bassiana* isolates and fungal references. Table S3: Chitinase KEEG validated specific counts for *B. bassiana* isolates and other fungal references. Figure S1: GenomeScope plot for eight *B. bassiana* isolates. Figure S2: Orthofinder overall distribution for eight *B. bassiana* genomes and two references (*B. bassiana* AREF 8028 and *C. militaris*), figure produced in R. Figure S3: OrthoVenn2 diagram for the *B. bassiana* isolates, and *B. bassiana* ARSEF 2860 and *C. militaris* reference genomes.
