# Supplementary material for: Genomic Characterization and Functional Description of Beauveria bassiana Isolates from Latin America
Source: J Fungi (Basel). 2023 Jun 29;9(7):711. doi: 10.3390/jof9070711 (PMC10381237; doi:10.3390/jof9070711)
Supplement: Supplementary file 1 [file jof-09-00711-s001.zip › Figures_S1_S3.pdf]

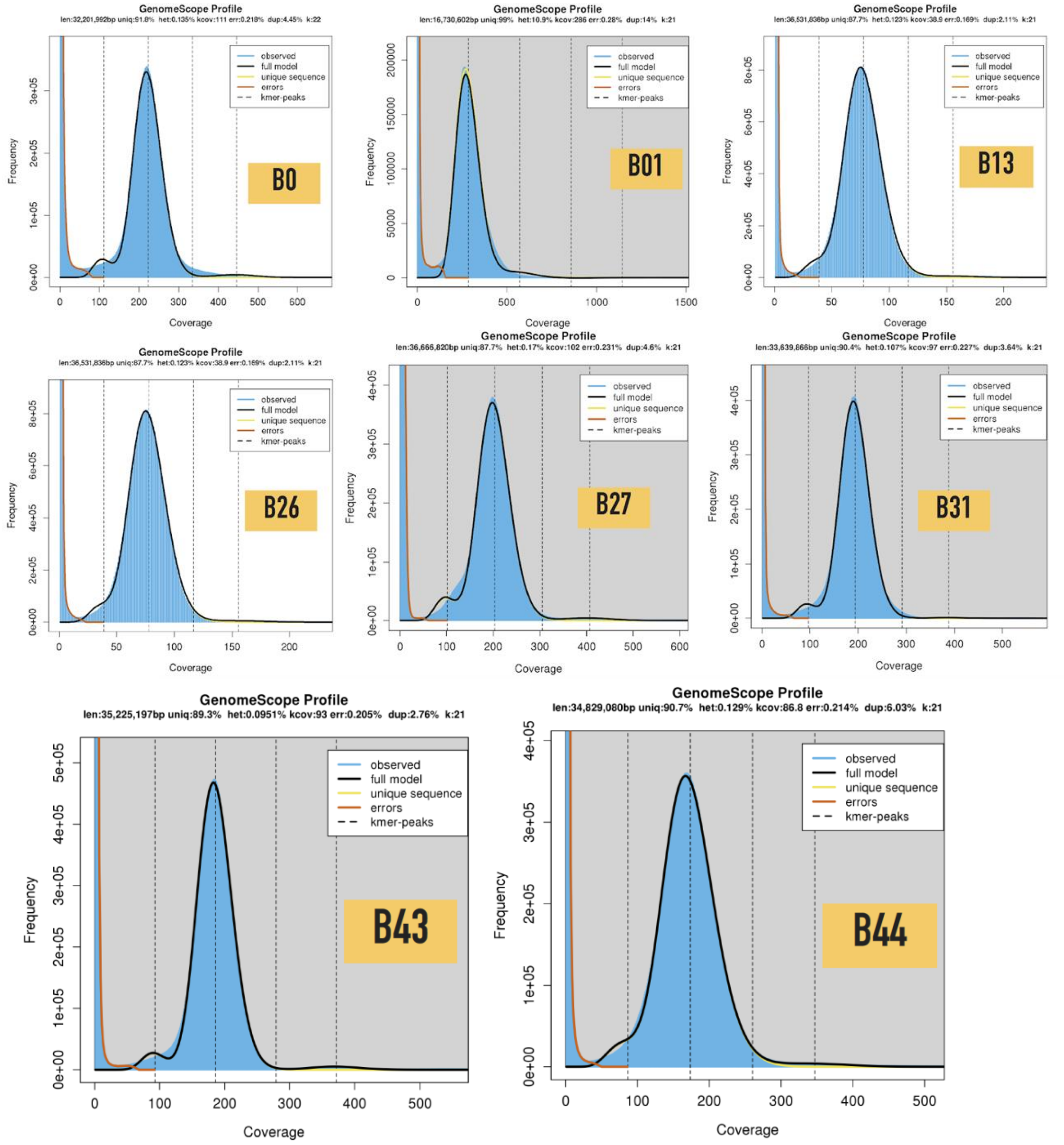

Supplementary Figure S1. GenomeScope plot for eight *B.bassiana* isolates. Each isolate (depicted with a yellow square) indicates a single peak demonstrating haploidy and an overall optimal kmer distribution under the blue curve.

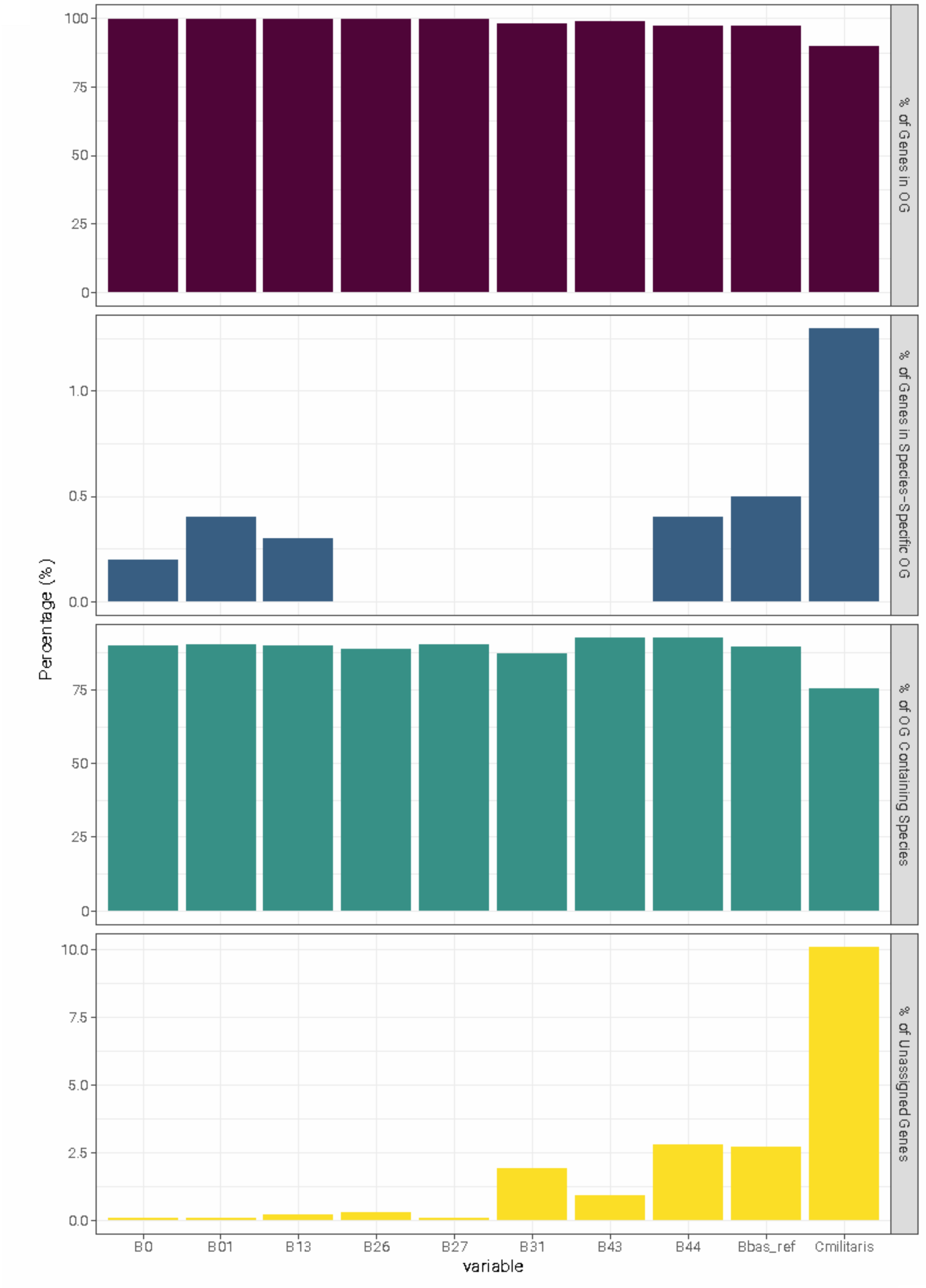

Supplementary Figure S2. Orthofinder overall distribution for eight *B. bassiana* genomes and two references (*B.bassiana AREF 8028* and *C. militaris*), figure produced in R.

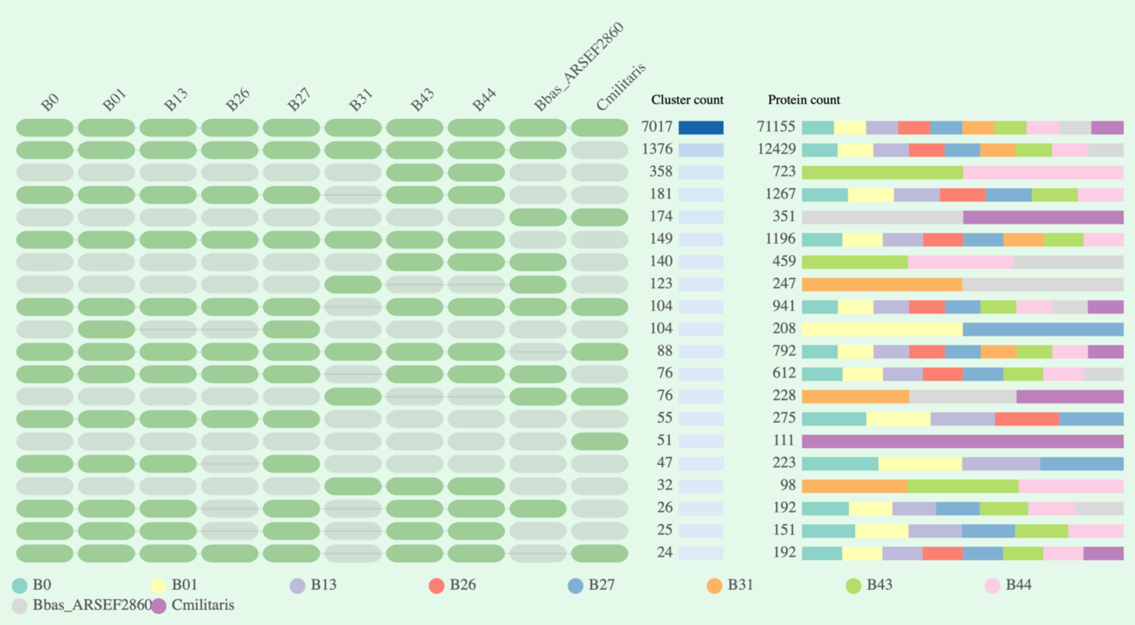

Supplementary Figure S3. OrthoVenn2 diagram for B.bassiana isolates, B.bassiana ARSEF 2860 and C.militaris reference genomes. Green circles represent orthogroups, colum "cluster count" indicates total cluster for species in the orthogroup and "Protein count" section depicts absolute protein abundance for each genome used as input (color codes at the bottom of the chart).
