# Supplementary material for: Genomic Characterization and Functional Description of Beauveria bassiana Isolates from Latin America
Source: J Fungi (Basel). 2023 Jun 29;9(7):711. doi: 10.3390/jof9070711 (PMC10381237; doi:10.3390/jof9070711)
Supplement: Supplementary file 1 [file jof-09-00711-s001.zip › Supplementary Material.pdf]

Supplementary Material

Table S1. SignalP gene Ids for each genome, individual gene numbers (per column)

Table S2. Counts for putative CAZymes for B. bassiana isolates and fungal references

Table S3. Chitinase KEEG validated specific counts for B. bassiana isolates and other fungal references

Figure S1. GenomeScope plot for eight B.bassiana isolates. Each isolate (depicted with a yellow square) indicates a single peak demonstrating haploidy and an overall optimal kmer distribution under the blue curve.

Figure S2. Orthofinder overall distribution for eight B. bassiana genomes and two references (B.bassiana AREF 8028 and C. militaris), figure produced in R.

Figure S3. OrthoVenn2 diagram for B.bassiana isolates, B.bassiana ARSEF 2860 and C.militaris reference genomes.
